# Blood or Fat? Differentiating Hemopericardium versus Epicardial Fat Using Focused Cardiac Ultrasound

**DOI:** 10.3390/diagnostics14080818

**Published:** 2024-04-15

**Authors:** Yuriy S. Bronshteyn, Nazish Hashmi, Jamie R. Privratsky, Atilio Barbeito

**Affiliations:** 1 Duke University Health System, Duke University School of Medicine, Durham, NC 27710, USA; 2 Durham Veterans Health Administration, Durham, NC 27705, USA

**Keywords:** pericardial effusion, epicardial fat pad, ultrasound, point-of-care ultrasound, echocardiography, hemopericardium

## Abstract

Basic point-of-care ultrasound of the heart—also known as Focused Cardiac Ultrasound (FoCUS)—has emerged as a powerful bedside tool to narrow the differential diagnosis of causes of hypotension. The list of causes of hypotension that a FoCUS provider is expected to be able to recognize includes a compressive pericardial effusion due to hemopericardium (blood in the pericardial sac). But hemopericardium can be difficult to distinguish from a more common condition that is not immediately life-threatening: epicardial fat. This paper reviews illustrative images of both epicardial fat and hemopericardium to provide practice guidance to the FoCUS user on how to differentiate these two phenomena.

Ultrasound in modern clinical practice can be broadly subdivided into two categories: consultative and point-of-care. Consultative ultrasound refers to the performance of an ultrasound exam requested by a patient’s primary treating provider but performed by a separate specialist team [1]. In contrast, point-of-care ultrasound (POCUS) is the use of ultrasound at the bedside by a patient’s primary treating provider to guide a procedure or answer a diagnostic question [1]. Further, within diagnostic POCUS of the heart two sub-categories exist: advanced and basic [1]. Because advanced cardiac POCUS employs advanced imaging modes such as color and spectral Doppler to quantitatively assess a broad spectrum of sonographically detectable cardiac pathology, it requires subspecialty-level training to achieve proficiency [1,2]. In contrast, because basic cardiac POCUS employs only greyscale imaging (B-mode +/− M-mode) to qualitatively screen for a short list of pathologies, it can be learned as part of routine training during residency/fellowship in any acute care specialty, such as internal medicine, critical care, emergency medicine, anesthesiology, etc. [3,4,5].

Given its relative accessibility, basic cardiac POCUS—also known as Focused Cardiac Ultrasound (FoCUS)—has emerged as a powerful tool that can help bedside clinicians to rapidly rule in and rule out multiple life-threatening conditions capable of causing hypotension [5]. Since the differential diagnosis of hypotension includes, among other things, a compressive pericardial effusion, basic POCUS providers are expected to be able to detect fluid around the heart, which can be either serous or sanguinous [6]. The latter situation, also known as hemopericardium, can be both immediately life-threatening and especially challenging to diagnose using FoCUS because blood in the pericardial sac can mimic tissue on ultrasound [7]. So, to accurately identify hemopericardium, clinicians practicing FoCUS should be able to recognize not only hemopericardium but also its most common mimic: an epicardial fat pad [7].

Epicardial fat is visceral fat deposited outside the heart and its thickness predicts both overall visceral adiposity and the likelihood of multiple chronic, inflammatory diseases [6,8]. Although epicardial fat is not immediately life-threatening, on ultrasound it is easily mis-identified as hemopericardium, with potential for severely inappropriate treatment of patients [7,9]. This is because hemopericardium and fat can appear similarly isoechoic on ultrasound, i.e., approximately as bright as solid tissue.

However, some ultrasound findings can help distinguish these two conditions, as shown in the attached figures and Supplementary Videos. First, the characteristic appearance of an epicardial fat pad is shown in two FoCUS views: the subcostal four-chamber (SC4C) in Figure 1 (Supplementary Video S1) and the parasternal long-axis (PLAX) in Figure 2 (Supplementary Video S2). Next, the same two FoCUS views are shown from a different patient where an epicardial fat pad and hemopericardium were present concurrently (Figure 3 and Figure 4).

As shown in the parasternal images (Figure 2 and Figure 4), epicardial fat is nearly always located anterior to the right ventricle (RV), whereas hemopericardium typically tracks posteriorly, between the left ventricle (LV) and descending thoracic aorta (DTA) [6,7]. Notably, differentiating blood from fat becomes more difficult when solely using the SC4C view (Figure 1 and Figure 3) because both pericardial blood and epicardial fat tend to occupy a similar location in this view between the liver and the right ventricle. Further, some additional criteria are used by echo-cardiologists to help differentiate fat and hemopericardium on ultrasound but in our experience these are challenging to operationalize for basic cardiac POCUS providers because these criteria are highly subjective: (i) fat is said to move synchronously with the heart whereas hemopericardium is said to move independently and (ii) fat is said to appear more echogenic (“brighter”) than myocardium whereas blood tends to be less echogenic (“darker”) [6]. 

These latter criteria illustrate the challenge of extrapolating heuristics developed for consultative ultrasound to the realm of POCUS, especially basic cardiac POCUS. Whereas echocardiology training requires one to three years dedicated to the interpretation of hundreds of cardiac ultrasound exams, basic cardiac POCUS training generally only requires performance of 25–50 FoCUS exams to fulfill nationally recognized minimum training standards [10,11,12,13]. So, although the diagnostic challenge of differentiating pericardial blood versus epicardial fat is well known among echocardiologists, in our experience it is poorly appreciated by basic cardiac POCUS providers [7]. Further, several popular diagnostic POCUS protocols used widely within emergency medicine, trauma surgery, and critical care currently employ a single-FoCUS view to screen for pericardial effusion: the SC4C view [14,15]. But, as shown in Figure 1, Figure 2, Figure 3 and Figure 4, in this single view, both pericardial blood and epicardial fat can look nearly indistinguishable. This conclusion is also supported by a study by Blaivas, et al., of diagnostic errors in the performance of the focused assessed with sonography in trauma (FAST) exam. In this study, 22 emergency medicine physicians (five attendings and 17 residents) who had completed structured training in the FAST exam each reviewed 11 SC4C views and were tested on their ability to determine the presence versus absence of pericardial effusion in each view. The authors found that the overall accuracy for discriminating epicardial fat from pericardial effusion was only 30%. Although accuracy improved slightly with increasing POCUS experience, even the physician sonographers with the highest levels of ultrasound experience achieved a specificity of only 49% [7].

Hence, we present these images and associated Supplementary Videos to help basic cardiac POCUS providers perform all of the following: (i) build their mental catalogues of important cardiac pathology; (ii) understand the importance of including a PLAX view when trying to differentiate epicardial fat versus blood; and (iii) appreciate that even with a PLAX view, it is sometimes difficult to differentiate blood versus fat at the bedside. In such cases, the FoCUS provider should consider the following additional diagnostic maneuvers: evaluation of the IVC and consultative imaging. First, because hemopericardium would be expected to quickly devolve into cardiac tamponade, FoCUS providers can try to differentiate blood from fat in the pericardial sac by screening for sonographic suggestion of tamponade. Whereas many sonographic signs of tamponade are, in our experience, challenging for FoCUS providers to detect accurately (e.g., right/left atrial compression during ventricular systole, right ventricular compression during ventricular diastole, and/or elevated respirophasic variation in trans-mitral/trans-tricuspid flow velocities), one sonographic clue to tamponade is well within the FoCUS skillset: evaluation of the IVC. The IVC in a spontaneously breathing patient without tamponade is expected to collapse at least 50% during brisk inspiration and/or during vigorous sniff [6]. In contrast, the IVC is plethoric (fixed and dilated) in approximately 90% of cases of cardiac tamponade [6]. But, notably, approximately 10% of cardiac tamponade cases lack IVC plethora (a phenomenon termed “low-pressure tamponade”) and may be difficult to diagnose with basic cardiac POCUS alone [6,16]. In such cases, the POCUS provider can consider two additional diagnostic tests if they are available and feasible: (i) consultative echocardiography and/or (ii) computed tomography (CT). On CT, attenuation is low for fat (negative Hounsfield units) and high for hemopericardium (positive units) [6]. 

## Figures and Tables

**Figure 1:**
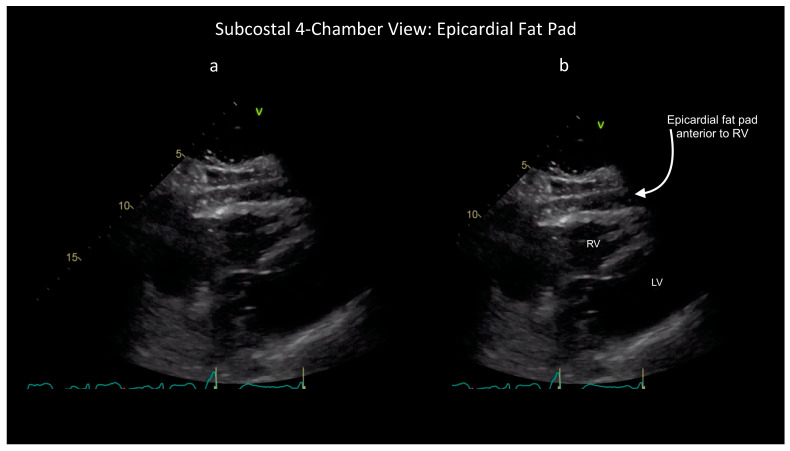
Unlabelled (**a**) and labelled (**b**) still image of a subcostal four-chamber view showing a large epicardial fat pad anterior to the right ventricle (RV). Left ventricle (LV) also visible. See also Supplementary Video S1.

**Figure 2:**
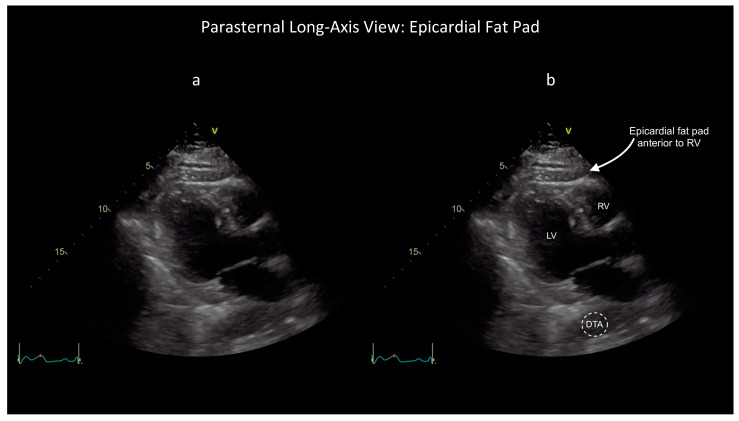
Unlabelled (**a**) and labelled (**b**) still image obtained as part of the same FoCUS exam that produced Figure 1 but now viewing the heart from the parasternal long-axis (PLAX) plane. Note the location of the epicardial fat pad anterior to the right ventricle (RV). In contrast, in the PLAX view, a pericardial effusion would be expected between the left ventricle (LV) and descending thoracic aorta (DTA). See also Supplementary Video S2.

**Figure 3:**
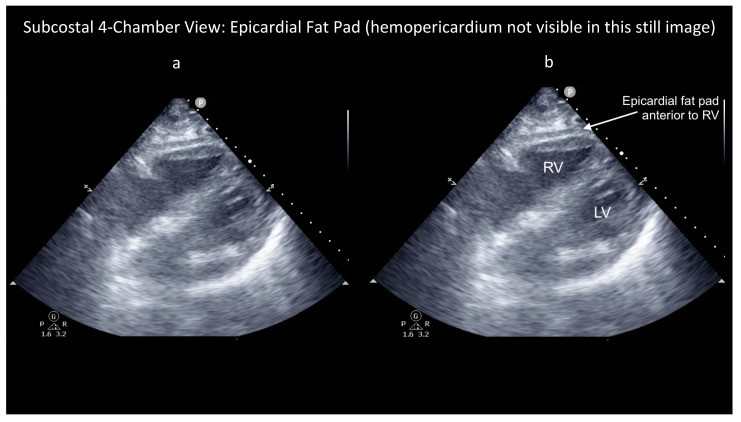
Unlabelled (**a**) and labelled (**b**) still image of a subcostal four-chamber (SC4C) view from a patient with concurrent epicardial fat pad and hemopericardium. This SC4C view shows a large epicardial fat pad anterior to the right ventricle (RV). Left ventricle (LV) also visible. Note that this patient’s concurrent pericardial effusion is not visible in this still image because it was only transiently visible in this view, specifically during ventricular systole (see Supplementary Video S3), but was readily apparent throughout the cardiac cycle in the parasternal long-axis view (see Figure 4 and Supplementary Video S4).

**Figure 4:**
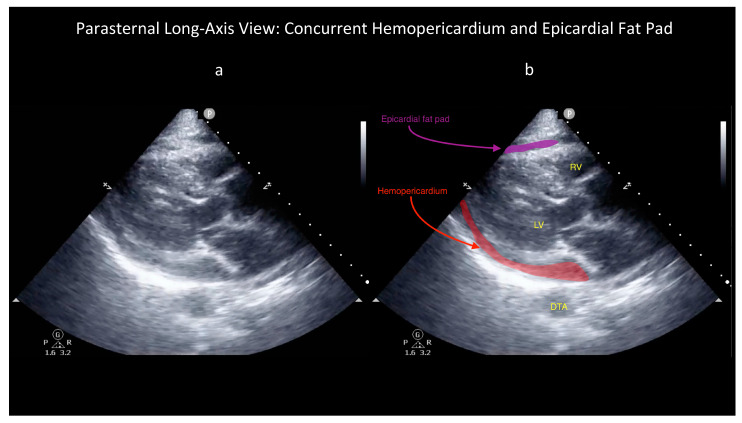
Unlabelled (**a**) and labelled (**b**) still image obtained as part of the same FoCUS exam that produced Figure 3 but now viewing the heart from the parasternal long-axis (PLAX) plane. Note the location of the epicardial fat pad anterior to the right ventricle (RV). Concurrently, this patient also had a moderate amount of hemopericardium visible in this view between the left ventricle (LV) and descending thoracic aorta (DTA) (see also Supplementary Video S4) whereas this blood is difficult to appreciate and/or differentiate from the patient’s epicardial fat in the subcostal four-chamber view (see Figure 3 and Supplementary Video S3).

## Data Availability

De-identified ultrasound clips are included as Supplementary Videos.

## Supplementary Materials

The following supporting information can be downloaded at: https://www.mdpi.com/article/10.3390/diagnostics14080818/s1, Supplementary Video S1: Subcostal four-chamber view demonstrating a prominent epicardial fat pad anterior to the right ventricle (see also Figure 1 for annotated still image from this video). Supplementary Video S2: Parasternal long-axis view demonstrating the same prominent epicardial fat pad anterior to the right ventricle seen in Supplementary Video S1 (see also Figure 2 for annotated still image from this video). Supplementary Video S3: Subcostal four-chamber view containing concurrently an epicardial fat pad and hemopericardium both anterior to the right ventricle (see also Figure 3 for annotated still image from this video). Supplementary Video S4: Parasternal long-axis view demonstrating the same prominent epicardial fat pad and hemopericardium seen in Supplementary Video 3, but now more clearly since the two findings are now visualized in diametrically opposite sides of the heart: the epicardial fat pad is anterior to the right ventricle whereas the hemopericardium is posterior to the heart, between the left ventricle and descending thoracic aorta (see also Figure 4 for annotated still image from this video).
